# The NAC domain-containing protein, GmNAC6, is a downstream component of the ER stress- and osmotic stress-induced NRP-mediated cell-death signaling pathway

**DOI:** 10.1186/1471-2229-11-129

**Published:** 2011-09-26

**Authors:** Jerusa AQA Faria, Pedro AB Reis, Marco TB Reis, Gustavo L Rosado, Guilherme L Pinheiro, Giselle C Mendes, Elizabeth PB Fontes

**Affiliations:** 1 Departamento de Bioquímica e Biologia Molecular/BIOAGRO, Universidade Federal de Viçosa, 36570.000, Viçosa, Minas Gerais, Brazil; 2 National Institute of Science and Technology in Plant-Pest Interactions. Universidade Federal de Viçosa, 36570.000, Viçosa, Minas Gerais, Brazil

**Keywords:** GmNAC6, Cell death, ER stress, osmotic stress, NRPs, N-rich proteins

## Abstract

**Background:**

The endoplasmic reticulum (ER) is a major signaling organelle, which integrates a variety of responses against physiological stresses. In plants, one such stress-integrating response is the N-rich protein (NRP)-mediated cell death signaling pathway, which is synergistically activated by combined ER stress and osmotic stress signals. Despite the potential of this integrated signaling to protect plant cells against different stress conditions, mechanistic knowledge of the pathway is lacking, and downstream components have yet to be identified.

**Results:**

In the present investigation, we discovered an NAC domain-containing protein from soybean, GmNAC6 (*Glycine max *NAC6), to be a downstream component of the integrated pathway. Similar to *NRP-A *and *NRP-B, GmNAC6 *is induced by ER stress and osmotic stress individually, but requires both signals for full activation. Transient expression of *GmNAC6 *promoted cell death and hypersensitive-like responses *in planta*. *GmNAC6 *and *NRPs *also share overlapping responses to biotic signals, but the induction of *NRPs *peaked before the increased accumulation of GmNAC6 transcripts. Consistent with the delayed kinetics of *GmNAC6 *induction, increased levels of *NRP-A *and *NRP-B *transcripts induced promoter activation and the expression of the *GmNAC6 *gene.

**Conclusions:**

Collectively, our results biochemically link GmNAC6 to the ER stress- and osmotic stress-integrating cell death response and show that GmNAC6 may act downstream of the NRPs.

## Background

Plants do not passively accept abiotic stresses, such as drought, salinity and variations of temperature, or biotic aggressors, such as viruses, bacteria, insects and fungi. To cope with these environmental stressors, plant cells have developed coordinated and integrated molecular networks for stress signal perception, transduction and adaptation mechanisms under adverse conditions of growth. In general, some adaptive cellular responses to a specific stress condition are interconnected with other environmental responses [1-3]. For instance, conditions of water stress result in both nutritional and osmotic stress, which can also be caused by salt stress. Similarly, increasing evidence in the literature has demonstrated the interconnection among the responses to pathogen attack and developmental signals [4-6]. In this complex interplay of physiological stresses, plant cells have evolved both anterograde and retrograde transduction pathways among the organelles to respond to environmental signals in an integrated and coordinated manner. One such major signaling organelle is the endoplasmic reticulum (ER), which integrates a variety of responses against stresses [7,8].

The ER is a multifunctional organelle that supports a series of basic cellular processes, such as protein folding and quality control, the maintenance of Ca^2+ ^balance and lipid biosynthesis. Any condition that disturbs ER homeostasis and ER function can induce stress in the organelle. In general, ER stress is initiated by an imbalance between the rate of protein synthesis and ER protein-processing activities. Under conditions in which the nascent, unfolded polypeptide influx into the lumen of the ER exceeds the folding and processing capacity of the organelle, unfolded proteins accumulate in the lumen of the ER and, in turn, trigger a cytoprotective pathway designated 'the unfolded protein response (UPR), which has been described in details in mammalian cells [for a review, see [9]]. To alleviate ER stress, the coordinated action of three UPR transducers, activating transcription factor 6 (ATF6), the inositol requiring kinase 1 (IRE1), and double-stranded RNA-activated protein kinase (PKR)-like endoplasmic reticulum kinase (PERK), leads to the activation of the following three types of cellular response: (1) the up-regulation of ER molecular chaperones, such as BiP (binding protein) and calnexin (CNX); (2) the attenuation of protein translation that is mediated by PERK through the phosphorylation of eukaryotic initiation factor 2α (eIF2α); and (3) the degradation of misfolded proteins by a process called 'ER-associated degradation' (ERAD). However, excessive or prolonged stress can lead to maladaptive responses and, ultimately, can activate apoptotic cell death to protect tissues from necrotic injury [9]. Recent studies have demonstrated that ER stress can also elicit an innate immunity defense to protect tissues in mammalian cells, and in plant cells, ER stress is linked to the host defense response to microbial infections [10-12]. Thus, in addition to the UPR, other signaling pathways radiate from the ER to the mitochondria, nucleus and possibly other organelles.

Recently, a global expression profiling on tunicamycin-induced and polyethylene glycol (PEG)-induced soybean leaves uncovered an ER stress- and osmotic stress-shared response represented by co-regulated genes that was found to be synergistically induced by both stresses [13,14]. Genes in this integrated pathway encode proteins with diverse roles, such as plant-specific development and cell death (DCD) domain-containing proteins, represented by the asparagine-rich proteins NRP-A and NRP-B, an ubiquitin-associated (UBA) protein homolog and NAC (*NAM, ATAF1, ATAF2 *and *CUC2*) domain-containing proteins. NAC proteins are plant specific transcriptional factors that are involved in a variety of developmental events as well as in biotic and abiotic stress responses [for a review, see [15]]. They comprise a large family of transcriptional regulator genes and, in the soybean genome, are represented by at least 101 sequences [16].

The N-rich protein (*NRP*) genes, which demonstrated the strongest synergistic induction, share a highly conserved C-terminal DCD domain in addition to a high content of asparagine residues at their more divergent N termini [13]. This structural organization places NRP-A and NRP-B in the subgroup I of plant-specific DCD-containing proteins [17]. We have recently demonstrated that both NRP-A and NRP-B induce a senescence-like response when ectopically expressed in soybean cells and tobacco leaves [13]. These studies have demonstrated that ER stress and osmotic stress pathways converge at the level of NRP gene activation to potentiate a cell death response. In fact, the combination of both stress signals intensifies the output of the different pathways upon NRP expression; therefore, NRPs serve as molecular links that integrate the ER stress and osmotic stress responses. This ER stress- and osmotic stress-integrating response has been designated as the NRP-mediated cell death signaling, which is synergistically activated by both stress signals. We have recently demonstrated that the transcriptional factor GmERD15 acts upstream of NRPs and activates the expression of NRP-A and NRP-B in response to osmotic stress and ER stress [18]. Although the integrated signaling pathway has the potential to accommodate general plant-specific adaptive responses, mechanistic knowledge of the pathway is lacking, and downstream components have yet to be identified. Here, we describe a member of the NAC domain-containing protein superfamily from soybean, GmNAC6 (*Glycine max *NAC6) as a possible downstream component of the pathway. In addition to being synergistically up-regulated by a combination of ER stress and osmotic stress signals, ectopic expression of *GmNAC6 *causes senescence-like responses *in planta*, a phenotype that resembles the NRP-mediated response. We also found that NRPs induce promoter activation and expression of GmNAC6 genes.

## Results

### GmNAC6 is induced by ER stress and osmotic stress individually but requires both signals for full activation

**Figure 1:**
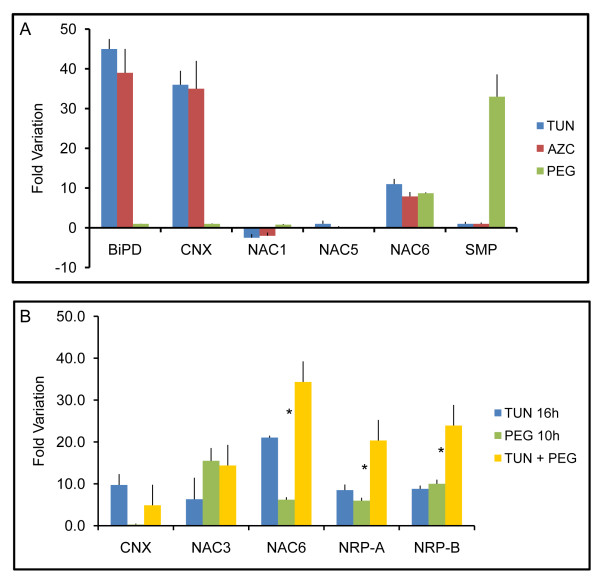
**The integration of ER-stress and osmotic-stress signals leads to full activation of GmNAC6**. A. The effect of PEG, tunicamycin or AZC on the expression of *GmNAC1, GmNAC5 *and *GmNAC6*. Three-week-old plants were treated with tunicamycin TUN (10 μg/ml, 24 h), PEG (MW:8000, 10%, 16 h) or AZC (50 mM, 16 h). The relative expression of representative genes of UPR (*BiPD *and *CNX*), osmotic stress-specific response (*SMP*) and senescence-associated soybean *GmNAC *genes (*NAC1, NAC5*, and *NAC6*) was determined by quantitative RT-PCR. Values for TUN are relative to the DMSO control treatment, and for PEG and AZC the values are relative to the H_2_O control; values represent the mean ± SD of three replicates from three independent experiments. B. The synergistic induction of GmNAC6 transcripts by a combination of PEG and tunicamycin treatments. Plants were treated with TUN (16 h) or PEG (10 h) alone or a combination of TUN + PEG. For the combined treatments PEG + TUN, the plants were pre-treated with tunicamycin for 6 h when PEG was added for an additional 10 h. RNA was isolated after the indicated time and quantified by real time RT-PCR, targeting the UPR-specific gene, *CNX*, the senescence-associated soybean genes, *GmNAC3 *and *GmNAC6*, and the integrated pathway genes, *NRP-A *and *NRP-B*. Asterisks indicate the position of additive responses. H_2_O and DMSO are control treatments for PEG and TUN, respectively. Values represent the mean ± SD of three replicates from three independent experiments.

To identify components of the ER stress- and osmotic stress-integrating NRP-mediated cell-death response, we searched among the co-regulated genes by both stresses [14] for those that were synergistically induced by both stress signals. In this regard, we focused our attention on an EST encoding a member of the NAC domain-containing protein family and extended our search to other members of the soybean NAC protein family. At least three members of the NAC domain-containing protein family from soybean --GmNAC1, GmNAC5 and GmNAC6-- have been associated with senescence or cell death [16]. However, only GmNAC6 was induced by the osmotic stress inducer, PEG, and the ER stress-inducing agents, tunicamycin (TUN) and L-azetidine-2-carboxylic acid (AZC), which cause protein misfolding in the ER by different mechanisms (Figure 1A). ER stress (*BiPD *and *CNX*) and osmotic stress (*SMP*) marker genes were included in the assay to ensure the efficiency of the tunicamycin and PEG treatments. The combination of ER stress and osmotic stress promoted a slightly more than additive effect on the accumulation of GmNAC6 transcripts in a fashion similar to the induction of the NRP-A and NRP-B genes (Figure 1B). These results indicate that the integration of ER-stress and osmotic-stress signals leads to the full activation of GmNAC6.

We also examined the induction of other members of the soybean NAC gene family, such as GmNAC3, which is up-regulated by PEG [16] and tunicamycin (Figure 1B) as well as during leaf senescence [17]. The combined exposure of soybean seedlings to both stress inducers, however, did not promote an additive or synergistic effect on the induction of GmNAC3. Taken together, these results substantiate the argument that GmNAC6, but not GmNAC3, may be a target of the NRP-mediated cell death signaling that integrates ER stress and osmotic stress responses.

### *GmNAC6 *promotes cell death in tobacco leaves and in soybean cells

**Figure 2:**
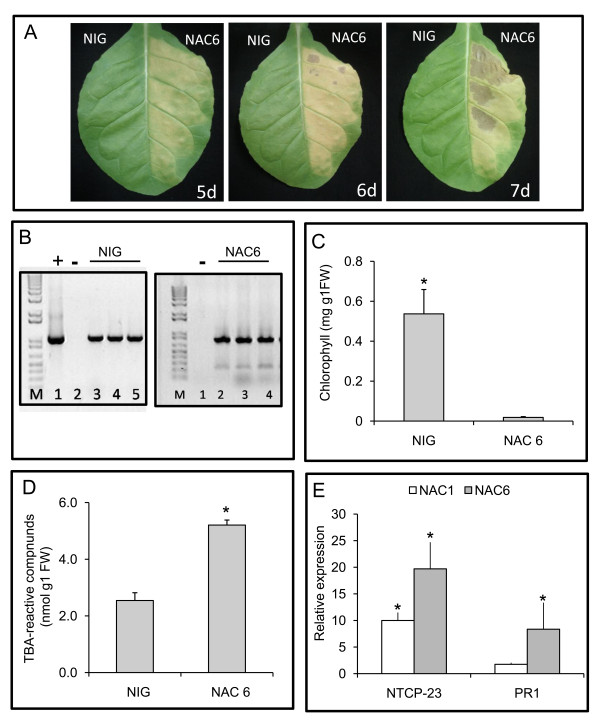
**GmNAC6 promotes cell death *in planta***. A. Three-week-old tobacco leaves were infiltrated with Agrobacterium cells carrying the 35S::YFP-NAC6 construct or an unrelated 35S::NIG construct. A. The yellowing phenotypes and necrotic lesions caused by GmNAC6 expression. Leaf sectors were infiltrated with the indicated Agro-inoculum and photographs were taken at 5 days (5 d), 6 days (6 d) and 7 days (7 d) post-Agro-inoculation. B. Transient expression of NIG and GmNAC6 genes in Agro-infiltrated leaf sectors at 5 days after Agroinfiltration. Semi-quantitative RT-PCR was on RNA of Agro-infiltrated leaf sectors with gene-specific primers, as indicated in the figure. C. Chlorophyll loss in the 35S::GmNAC6-infiltrated sectors. Total chlorophyll was determined from the leaf sectors Agro-infiltrated for 5 days with the samples in (A). The values are given as mean ± SD from three replicates. D. Lipid peroxidation induced by GmNAC6 expression. The lipid peroxidation in the 5-d-infiltrated leaf sectors from (A) was monitored by determining the level of TBA-reactive compounds. The values are given as mean ± SD from three replicates. Asterisks indicate values significantly different from the control treatment (p < 0.05, Tukey HSD test). E. The induction of the senescence-associated gene, NTCP23, and pathogenesis-related gene 1, PR1, by GmNAC6 expression. Total RNA was isolated from 5-day-infiltrated leaf sectors that were infiltrated with 35S::GmNAC6 (gray bars) or 35S::GmNAC1 (white bars), and the gene induction was monitored by quantitative RT-PCR using gene-specific primers. Values are relative to the control treatment (NIG infiltration) and asterisks indicate statistic differences (p < 0.05, Tukey HSD test).

We have recently demonstrated that the integrated pathway transduces a programmed cell death (PCD) signal generated by ER- and osmotic-stresses that results in the appearance of markers associated with leaf senescence [13]. To assess whether GmNA6 is involved in cell death, we assayed for hallmarks of leaf senescence, such as chlorotic lesions, chlorophyll loss, lipid peroxidation and the induction of senescence-associated genes in tobacco leaf sectors infiltrated with Agrobacterium carrying a 35S::GmNAC6 construct. After five days post-infiltration, the leaf sectors expressing *GmNAC6 *displayed a chlorotic phenotype with necrotic lesions that rapidly evolved to intense necrosis at seven days post-infiltration as a result of massive cell death; this observation was in marked contrast with the expression of an unrelated *NIG *gene [19] used as a negative control (Figure 2A). We also noticed that the GmNAC6-induced chlorotic phenotype appeared more rapidly than that promoted by expression of *NRP-B *gene (compare Figure 2A and Additional file 1). In fact, under similar conditions, the symptoms induced by NRP-B expression were first visible at 8 days post-Agro-infiltration when an increase in membrane ion leakage of the NRP-B Agroinfiltrated leaves was also observed (Additional file 2A).

The expression of *GmNAC6 *(Figure 2B) promoted chlorophyll loss in the Agroinfiltrated sectors (Figure 2C), an increase in membrane ion leakage of Agroinfiltrated leaves (Additional file 2B) and a significant increase in lipid peroxidation (Figure 2D) at five days after infiltration. The latter was examined by measuring the accumulation of thiobarbituric acid (TBA)-reactive compounds, which was clearly enhanced in the 35S::*GmNAC6 *Agro-inoculated leave sectors, when compared with the leaf slices that were Agro-inoculated with the control *35S::NIG *gene. These TBA-reactive compounds are products of senescence-associated lipid peroxidation, a process that results in the generation of reactive oxygen species (ROS) and chlorophyll loss [20].

We further confirmed the GmNAC6-induced senescence-like phenotype by monitoring the expression of the senescence-associated gene markers, *NTCP-23 *(AB032168, called CP1 in [13], which has been shown to be up-regulated in association with tobacco leaf senescence [13,21], and the pathogenesis-related gene 1 [*PR1*, [22]], by quantitative RT-PCR. The expression of GmNAC6 promoted an enhanced accumulation of NTCP-23 and PR1 transcripts (Figure 2E). GmNAC1, which has also been shown to be associated with senescence in soybean [16], induced the expression of *NPCP-23 *and, to a much lesser extent, *PR1 *when transiently expressed in tobacco leaves, demonstrating the effectiveness of the assay in this heterologous system. Taken together, these results indicate that *GmNAC6 *expression induces a senescence-like response in tobacco leaves.

**Figure 3:**
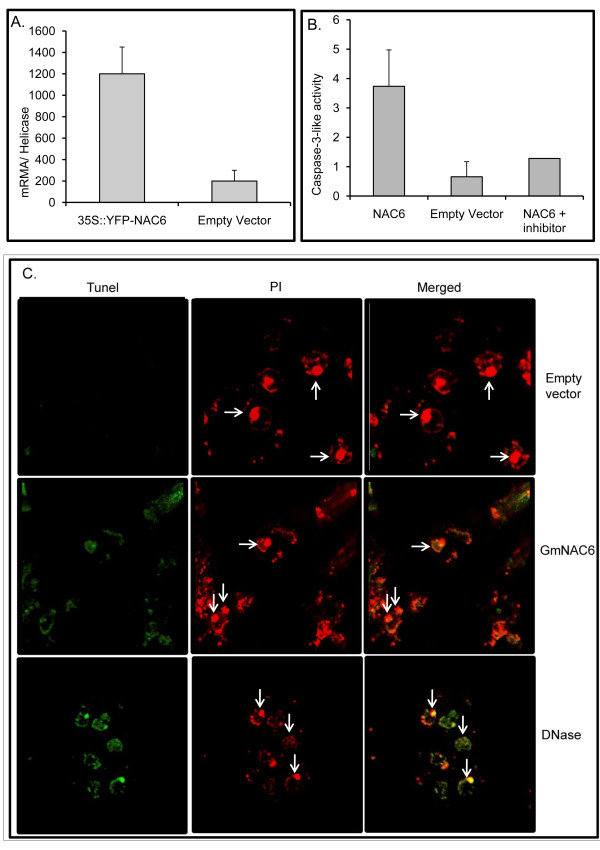
**The transient expression of *GmNAC6 *in leaf soybean protoplasts induces cell death**. Transient expression of *GmNAC6 *in protoplasts. Soybean protoplasts were electroporated with the 35S::YFP-NAC6 construct or the empty vector, and the expression of *GmNAC6 *and YFP-GmNAC6 was monitored by quantitative RT-PCR 36-h after electroporation. Values of expression were calculated using the 2^-ΔCt ^method and helicase as endogenous control. Values represent the mean ± SD of three replicates. B. Caspase-3-like activity. Total protein was extracted from GmNAC6-electroporated protoplasts after 36 h, and caspase 3-like activities were monitored with a DEVD-pNA substrate in the absence and presence of a specific inhibitor. Values represent the mean ± SD of three replicates. C. DNA fragmentation promoted by *GmNAC6 *expression. Cells were sampled 36-h post-electroporation of soybean protoplasts with empty vector or *GmNAC6 *expression cassette, submitted to TUNEL labeling and examined by confocal microscopy. The cells were also counterstained with propidium iodide (PI) and examined for red fluorescence at 632 nm. Arrows indicate some nuclei. Merged is an overlay of the fluorescent image of TUNEL labeling with PI staining cells to facilitate the identification of TUNEL-positive nuclei. As a positive control, untransfected cells were also treated with DNase.

Because NRPs, effectors of the ER stress and osmotic stress-integrating cell death response, have also been shown to induce cell death when transiently expressed in soybean cells, we examined whether GmNAC6 could induce the activity of caspase 3-like and DNA fragmentation in the endogenous system. The transient expression of *GmNAC6 *was driven by the 35S promoter in soybean protoplasts and was measured by RT-PCR, relative to a helicase marker to control for any variation in the transformation efficiency (Figure 3A). The caspase 3-like activity in total protein extracts from GmNAC6-overexpressing soybean cells was 3.62-fold higher than in extracts from protoplasts transformed with the empty vector (Figure 3B). We also used the terminal deoxynucleotidyltransferase-mediated dUTP nick end labeling (TUNEL) technique to measure fragmentation of DNA in individual cells 36 hours post electroporation (Figure 3C). After TUNEL labeling, the fomaldehyde-fixed and permeabilized semi-protoplasted leaf cells were also counterstained with propidium iodide (PI). Under these conditions, PI stained all cells and the red fluorescence signal concentrates in the nucleus as we treated the samples with RNase (Figure 3C, empty vector, see arrows). The nuclei of control cells transformed with the empty vector fluoresced intensely with propidium iodide (PI, red) and exhibited only TUNEL-negative nuclei (panel empty vector). In contrast, the *GmNAC6*-expressing samples had TUNEL positive nuclei that showed the same extent of staining as *NRP-B *(data not shown) and DNase treated positive controls. Merged is an overlay of the fluorescent image of TUNEL labeling with PI staining cells to facilitate the identification of TUNEL-positive nuclei. From two independent experiments, approximately 21% ± 1.5 of the semi-protoplasted leaf cells transformed with 35S::GmNAC6 had TUNEL- positive nuclei. Very likely the low efficiency of protoplasts transformation may account for the relatively low percentage of TUNEL positive nuclei in protoplasts electroporated with 35S::GmNAC6. Because caspase 3-like activity and DNA fragmentation have been described as biochemical markers associated with programmed cell death in soybean suspension cells [13], our results are consistent with an involvement of GmNAC6 in cell death events.

### NRPs and GmNAC6 are coordinately induced by biotic stresses but with different kinetics

**Figure 4:**
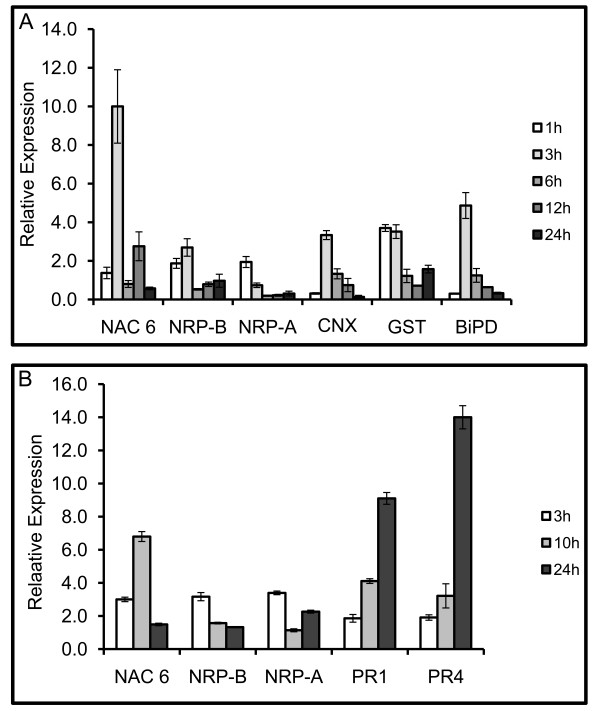
**Time course of GmNAC6 induction by biotic stress signals**. A. GmNAC6 is induced by treatment with cell wall-degrading enzymes (CDE). Soybean leaves were infiltrated with CDE, as described in the Methods, for the indicated times. Total RNA was isolated from the infiltrated sectors, and the relative expression of GmNAC6 (NAC6), UPR-specific gene markers (CNX and BiP) and integrated pathway genes (NRP-A, NRP-B, and GST) was determined by quantitative RT-PCR. B. Up-regulation of GmNAC6 by the hypersensitive response. Soybean leaves were inoculated with *Pseudomonas syringae patovar tomato *(*P.st*.) for the indicated period of time. The relative expression of GmNAC6, the integrated pathway genes (NRP-A and NRP-B) and pathogenesis-related genes (PR1 and PR4) was determined by quantitative RT-PCR.

The activation of the NRP-mediated senescence-like response is not specific to ER stress or osmotic stress but is, rather, a shared branch of general environmental adaptive pathways. In fact, *NRPs *are also induced by other abiotic and biotic signals, such as drought and pathogen-incompatible interactions [23,24]. As a putative component of NRP-mediated signaling, we examined whether *GmNAC6 *is induced by biotic signals as well (Figure 4). We first treated soybean leaves with cell wall-degrading enzymes (CDE), which mimic bacterial pathogen attack and induce a defense response [25], and then we inoculated soybean leaves with the incompatible bacterium, *Pseudomonas syringae patovar tomato *(Additional file 3), as our experimental system. Levels of GmNAC6 mRNA were examined at various times after treatment with CDE and inoculation with the bacterial pathogen (Figures 4A and 4B). As positive controls in the CDE treatments, we also examined the expression of the ER-resident molecular chaperones, binding protein (BiP) and calnexin (CNX), which have previously been demonstrated to be induced by CDE [10], and the glutathione-S-transferase (GST) gene that is also co-regulated by ER stress and osmotic stress [14] in the same fashion as NRPs and GmNAC6 (Figure 4A). For assaying the effectiveness of the incompatible bacterium, *Pseudomonas syringae patovar tomato*, in soybean, we examined the induction of the pathogenesis-related genes, *PR1 *and *PR4 *(Figure 4B). As with the *NRPs*, both of the treatments promoted the induction of *GmNAC6 *but with slightly different kinetics. The CDE treatment (Figure 4A) and bacterial inoculation (Figure 4B) resulted in increased NRP-A and NRP-B transcript levels as early as 1 hour and 3 hour, respectively, after the treatments. In contrast to the rapid induction of NRPs, the induction of *GmNAC6 *occurred with delayed kinetics, similar to the ER-resident chaperones, *BiP *and *CNX*, (Figure 4A) and the pathogenesis-related genes, *PR1 *and *PR4 *(Figure 4B). The induction of *GmNAC6 *by the CDE treatment and by the inoculation of the incompatible bacterium was first detected 3 h after the treatments. The GmNAC6 transcripts reached maximal accumulation at 10 h after inoculation of the soybean leaves with the incompatible bacterium (Figure 4B). These results indicate that *NRP-A *and *NRP-B *induction precedes the increased expression of *GmNAC6*.

### NRP-A and NRP-B induce the expression of the *GmNAC6 *gene

**Figure 5:**
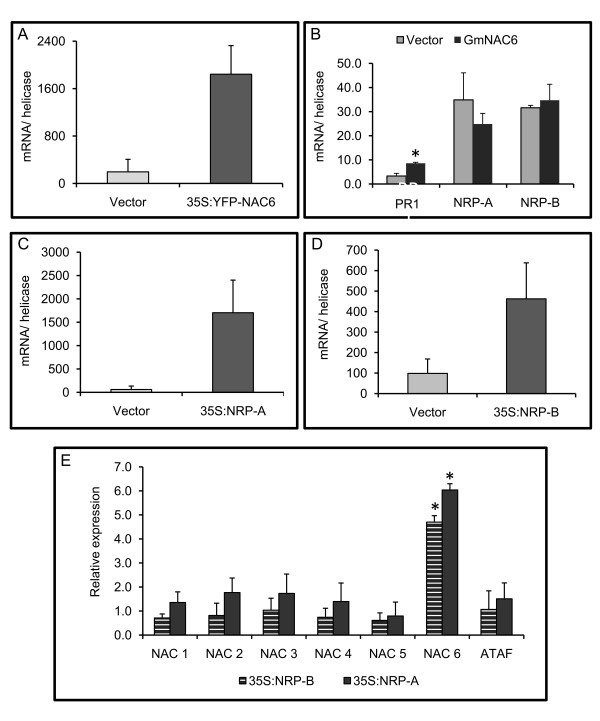
**The transient expression of NRP-A and NRP-B induces GmNAC6 expression**. A. The expression of GmNAC6 in soybean protoplasts from suspension cells. Soybean protoplasts were electroporated with the 35S::YFP-NAC6 construct or the empty vector, and the expression of GmNAC6 and YFP-GmNAC6 was monitored by qRT-PCR. The values represent the mean ± SD of three replicates from two independent experiments. B. The transient expression of YFP-GmNAC6 does not impact NRP-A or NRP-B transcript accumulation. Soybean protoplasts were electroporated with the 35S::YFP-NAC6 construct (dark gray) or the empty vector (light gray), and the expression of NRP-A and NRP-B was determined by qRT-PCR. The values represent the mean ± SD of three replicates from three independent experiments. Asterisks indicate values significantly different from the control treatment (p < 0.05, Tukey HSD test). C and D. The expression of NRP-A and NRP-B in soybean protoplasts. Plasmids containing NRP-A (C) or NRP-B (D) expression cassettes were electroporated into soybean protoplasts, and the transient gene expression was monitored by quantitative RT-PCR as in (A). E. The specific induction of GmNAC6 by NRP-A or NRP-B transient expression. Plasmids containing NRP-A (light gray) or NRP-B (dark gray) expression cassettes were electroporated into soybean protoplasts, and the relative expression of NAC genes was monitored by qRT-PCR. The relative quantitation of expression was calculated using 2^-ΔΔCt ^method. The values are relative to the control treatment (empty vector), and asterisks indicate those significantly different from the control treatment (p < 0.05, Tukey HSD test).

The coordinated synergistic induction of *GmNAC6 *by osmotic stress and ER stress, along with its capacity to promote NRP-like senescence phenotypes and PCD-like responses in plants, linked GmNAC6 to the ER stress and osmotic stress-integrating NRP-mediated signaling. To position GmNAC6 in this pathway, we examined the expression of *GmNAC6 *and *NRPs *in response to each other. The genes *GmNAC6 *(Figure 5A), *NRP-A *(Figure 5C) and *NRP-B *(Figure 5D) were placed under the control of the 35S promoter and overexpressed in soybean protoplasts derived from cultured cells. We first analyzed the kinetics of *NRPs *and *GmNAC6 *induction in response to the plant cell wall-degrading enzymes (CDE) used during the protoplasting procedure (Additional file 4). NRP-B transcripts were rapidly and transiently induced by CDE treatment, whereas the kinetics of GmNAC6 induction was delayed. Consistent with the delayed kinetics of the *GmNAC6 *induction by CDE treatment and physiological stresses, we found that the transient expression of *GmNAC6 *in soybean protoplasts did not result in the increased accumulation of *NRP *transcripts (Figure 5B). In contrast, the transient expression of both *NRP-A *or *NRP-B *induced *GmNAC6 *expression (Figure 5E). The increased accumulation of GmNAC6 transcripts by NRPs was a specific, rather than a general, phenomenon because the transient expression of *NRP-A *or *NRP-B *did not promote an up-regulation of other members of the soybean NAC domain-containing protein family. These results demonstrate that NRPs can induce GmNAC6, but GmNAC6 cannot induce NRPs.

### Transient expression of NRPs activates the GmNAC6 promoter in soybean cells

**Figure 6:**
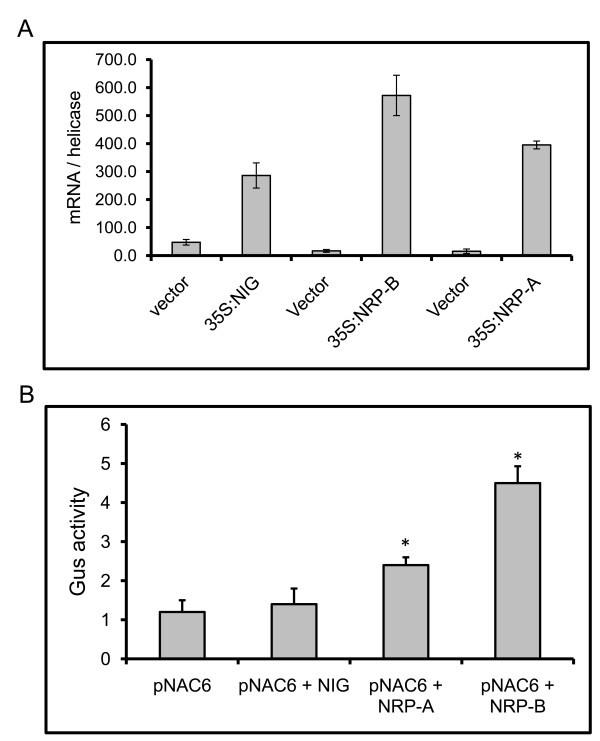
**NRP-A and NRP-B induces GmNAC6 promoter activation**. A. The transient expression of NIG, NRP-A and NRP-B in soybean protoplasts from suspension cells. Plasmids carrying 35S::NIG, 35S::NRP-A or 35S::NRP-B expression cassettes or empty vector were electroporated into soybean protoplasts, and the efficiency of transfection was monitored by determining the transient expression (qRT-PCR) 36 h after electroporation. B. The transient expression of NRP-A and NRP-B in soybean protoplasts activates a GmNAC6 promoter::β-glucuronidase gene. Soybean protoplasts were co-electroporated with plasmids carrying a GmNAC6-promoter::β-glucuronidase gene and either 35S::NIG, 35S::NRP-A or 35S::NRP-B DNA constructs, or the empty vector (pNAC6). After 36 h, the β-glucuronidase activity (nmol/min/mg protein) was measured in the total protein extracts of transfected soybean cells. The values represent the mean ± SD of five replicates from three independent experiments. Asterisks indicate mean values statistically different from the control treatment.

We next examined whether the observed activation of *GmNAC6 *by NRPs was at the transcriptional level by a transient expression assay in soybean protoplasts with an NRP-B promoter::β-glucuronidase (GUS) reporter construct. In this construct, a 5'-flanking sequence fragment of *NRP-B *(up to position -1000, relative to the translational initiation codon) was used to drive *GUS *expression. Because *GmNAC6, NRP-B and NRP-A *are transiently induced during the protoplast preparation by CDE (Additional file 4) and wounding [18], we measured the activity of the reporter gene at 36-h after transfection, when the expression of NRPs returned to basal levels and the accumulation of GUS driven by the CDE- and wounding-induced GmNAC6 promoter was expected to decline to lower levels (see Additional file 4). Under these conditions, the transient expression of *NRP-A *and *NRP-B *in soybean protoplasts (Figure 6A) resulted in increased reporter gene expression (Figure 6B), indicating that the control of *GmNAC6 *expression by NRP-B and NRP-B occurs, at least in part, at the transcriptional level. We also included the expression of the unrelated *NIG *gene in the assay as a negative control for specific promoter activation.

## Discussion

**Figure 7:**
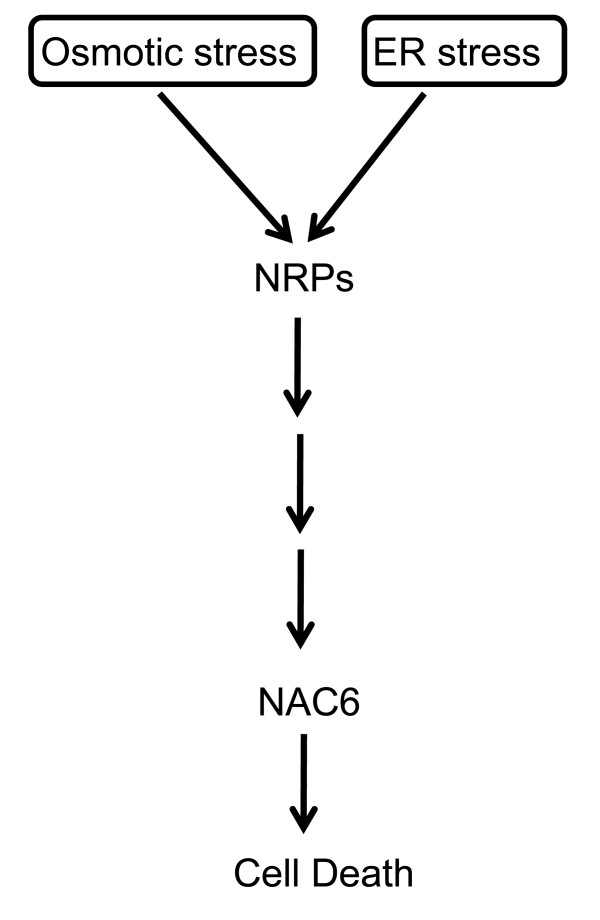
**The osmotic-stress and ER-stress signal-integrating pathway**. ER stress and osmotic stress activate two independent signaling pathways (1 and 2), which converge on NRP-A and NRP-B expression to activate an osmotic- and ER-stress integrating pathway, also called the integrated pathway. The enhanced accumulation of membrane-associated NRPs activates a cascade to induce the expression of the nuclear transactivator, NAC6, which, in turn, promotes cell death.

In contrast to the UPR, the NRP-mediated cell death signaling pathway is a plant-specific ER-stress cell-death response that communicates with other environmental stimuli through shared components. In fact, osmotic stress also activates the transduction of a cell death signal through NRPs. The convergence of both stress signals on NRP expression in a synergistic manner allows the transfer of information between these two distinct stress response pathways to potentiate a cell death response. Therefore, the integration of the ER stress and osmotic stress signals into a circuit of cell death occurs through the activation of NRP-mediated signaling. This cell death integrated pathway has emerged as a relevant adaptive response of plant cells to multiple environmental stimuli. Nevertheless, knowledge about this signaling pathway is limited to the identification of NRP as a crucial mediator of the cell death response and GmERD15 as a transcriptional factor that activates NRPs expression. Here, we describe a member of the NAC domain-containing protein family from soybean, GmNAC6, that may act downstream of NRP-A or NRP-B in the integration of the ER-stress and osmotic-stress cell death signals. GmNAC6 was linked to NRP-mediated cell death signaling based on three criteria. First, we showed that GmNAC6 expression was up-regulated by ER stress and osmotic stress individually, but when combined, the two stress signals promoted a synergistic accumulation of GmNAC6 transcripts. The synergistic induction of gene expression by the combination of ER stress and osmotic stress inducers is one of the criteria that link target genes to the ER stress- and osmotic stress-integrating pathway. Second, similar to the NRPs, the transient expression of GmNAC6 induced a senescence-like response in tobacco leaves and a cell death response in soybean cells. Lastly, the ectopic expression of *NRP-A *and *NRP-B *in soybean cells promoted the activation of the GmNAC6 promoter and the induction of *GmNAC6 *expression. Collectively, these results position GmNAC6 downstream of the NRPs in the ER stress- and osmotic stress-integrating pathways (Figure 7). However, whether GmNAC6 is linearly coupled to the NRPs in the integrated pathway is still a matter of debate.

NRPs and GmNAC6 were also induced by biotic signals, such as incompatible interactions and CDE treatment, but with different kinetics (Figure 4). While NRPs were rapidly induced by both treatments, increased accumulation of GmNAC6 transcritps occurred with a delayed kinetics. These data were consistent with the delayed induction of GmNAC6 during protoplast preparation, which generates similar signal as the CDE treatment. Therefore, an increased accumulation of NRP-B transcripts preceded the induction of *GmNAC6 *expression, which supports the argument that GmNAC6 acts downstream of NRPs. This interpretation is further substantiated by the observation that, in our experimental tobacco leaf transient expression system, GmNAC6-induced cell death occurred more rapidly than NRP-mediated cell death, as it would be expected from effectors acting downstream of NRPs in the cell death signaling pathway.

We found that NRP-B in soybean protoplasts induced *GmNAC6 *expression and activated GmNAC6 promoter. Whether the NRP-mediated up-regulation of *GmNAC6 *expression is a direct result of NRPs transactivation of gene expression or a secondary effect of signal transduction mediated by NRP it remains to be determined. Our data favor the latter hypothesis, as we have previously shown that soybean NRPs are localized in the cytoplasm in association with the plasma membrane (13). The Arabidopsis NRP homolog is also a cytosolic protein, but is translocated to the mitochondria under stresses conditions [26]. We don't know whether the soybean NRPs also share a stress-mediated mitochondrial compartmentalization, but we have failed to demonstrate a nuclear localization of NRP-B as it would be expected for a transcriptional activation function. Sequence analysis of 1-kb 5'flanking sequences of *GmNAC6 *revealed some conserved motifs of most eukaryotic promoters, such as a TATA box (Additional file 5 in pink) and an inverted CCAAT box (in bold), in addition to several potential regulatory elements of plant promoters, potentially involved in response to events of cell death or to osmotic stress and drought. These include an ABA-responsive element, the motif III of rice RAB16b gene1 (in purple), a binding site (in green) of OsBIHD1, a rice BELL homeodomain transcription factor involved in disease resistance, four putative elements (NGATT, in red) for the cytokinin-regulated transcription factor ARR1 and two binding sites (in blue) found in the ERD1 gene, involved in response to dehydration stress and dark-induced senescence. These putative cis-regulatory elements on the GmNAC6 promoter illustrate potential sites for assembly of transcription factors, which might constitute targets of the NRP-mediated stress-induced cell death response.

The evidence that *NRPs *and *GmNAC6 *were also induced by biotic signals implies that the NRP-mediated cell death signaling is a general adaptive response of plants. The protective role of the induction of PCD by pathogens during incompatible interactions, a phenomenon well documented in plants, restricts the pathogenic attack to the inoculated cells [27]. The rapid induction of *NRP *genes by incompatible interactions indicates that the NRP-mediated induction of PCD may be part of the hypersensitive response. Consistent with this hypothesis, the transient expression of GmNAC6 in tobacco leaves promoted the induction of the pathogenesis-related gene 1, PR1 and caused necrotic lesions.

In addition to being induced by ER stress and osmotic stress, NRP-mediated signaling is also induced by drought [18]. These abiotic stress signals induce a shared cell death response through NRPs. While the ER stress branch of the response is distinct from the molecular chaperone-induced branch of UPR [13], we previously showed that the osmotic stress branch of the response may be acid abscisic (ABA)-dependent [16]. In fact, both NRP-B and GmNAC6 are induced by ABA. Furthermore, evidence in the literature has demonstrated an antagonistic effect of ABA on salicylic acid (SA)-dependent defense pathways [28,29]. Thus, it may be possible that the activation of NRP-mediated signaling leads to enhanced SA-mediated responses, as shown by the induction of *PR1 *and hypersensitive response-like phenotypes, and acts antagonistically to suppress ABA-mediated responses. As ABA is a central regulator of plant adaptation to drought [30,31] and plays a crucial role in the regulation of transpirational water loss [32], it would be interesting to investigate whether an inactivation of the NRP-mediated cell death response would promote tolerance to dehydration.

## Conclusions

We have previously demonstrated that the integration of the ER stress and osmotic stress signals into a circuit of cell death occurs through the activation of NRP-mediated signaling pathway [13,14]. Expression of NRPs has been shown to be regulated by GmERD15, an ER- and osmotic-stress-induced transcriptional factor [18]. Here, we provided several lines of evidence that link the NAC domain-containing protein GmNAC6 to the NRP-mediated cell death response. Like *NRPs, GmNAC6 *is synergistically activated by a combination of ER stress and osmotic stress signals and induces a senescence-like response *in planta *and cell death in soybean protoplasts. *NRPs *and *GmNAC6 *are coordinately regulated by a variety of biotic and abiotic stresses but induction of *NRPs *precedes the up-regulation of *GmNAC6*. Consistent with this early induction kinetics, expression of NRPs activates the GmNAC6 promoter and induces *GmNAC6 *expression. Collectively, these results suggest that GmNAC6 may act downstream of NRPs in the ER stress- and osmotic stress-integrating cell death response (Figure 7). This interpretation is further substantiated by the observation that transient expression of GmNAC6 in tobacco leaves induces a more rapid cell death response than that mediated by NRP expression, as it would be expected from effectors acting downstream of NRPs in the cell death signaling pathway. However, whether GmNAC6 is linearly coupled to NRP in the integrated pathway remains to be determined.

## Methods

### Plasmid constructs

The clone 35S::YFP-NAC6, harboring the NAC6 cDNA fused to yellow fluorescent protein (YFP) under the control of the 35S promoter, has previously been described [16]. Similarly, the clones 35S::NRP-A, 35S::NRP-B [13] and 35S::NIG [19], containing the respective cDNAs under the control of the promoter 35S, have already been described.

### Plant growth, soybean suspension cells and stress treatments

Soybean (*Glycine max*) seeds (cultivar Conquista) were germinated in soil and grown under greenhouse conditions (an average temperature of 21°C, max. 31°C, min. 15°C) under natural light, 70% relative humidity, and approximately equal day and night length. Two-weeks after germination, the seedlings were transferred to 2 mL of 10% (w/v) polyethylene glycol (PEG; MW 8000, Sigma), 10 μg/mL tunicamycin (Sigma; DMSO, as control) or 50 mM L-azetidine-2-carboxylic acid (AZC, Sigma) solutions. After 8 h of treatment, the leaves were harvested, immediately frozen in liquid N_2 _and stored at -80°C until use. Alternatively, the aerial portions of three-week-old plants were excised below the cotyledons and were directly treated with tunicamycin or PEG as described [13,14]. Each stress treatment and RNA extraction was replicated in three independent experiments.

For the incompatible interaction experiments, soybean plants in the developmental stage VC [completely expanded unifoliate leaves, as described in the phenologic scale of Fehr and Caviness, [33]] were challenged with *Pseudomonas syringae patovar tomato*. The bacterial cells were grown at 28°C in 523 medium [34]. After centrifugation, the bacterium culture was resuspended in 10 mM MgCl_2 _to an O.D_600 nm _of 0.2, corresponding to approximately 1 × 10^7 ^cells/mL [35]. Soybean leaves were inoculated with the bacterial suspension in the abaxial epidermis of the leaves by using a lightly pressured syringe. At the intervals indicated in the figure legends, the leaf tissue was frozen in liquid nitrogen and stored at -80°C until use.

The treatment of soybean leaves with cell wall-degrading enzymes (CDEs) was performed as previously described [10]. Briefly, soybean leaves at the VC stage [32] were infiltrated with an enzymatic solution (0.4% cellulase, 0.2% macerozyme, 0.6% mannitol, and 20 mM MES, pH 5.5) or with buffer alone (0.6% mannitol and 20 mM MES, pH 5.5) as a control. Approximately 3, 10 or 24 h after inoculation, infiltrated leaves were harvested for analysis.

### Real-time RT-PCR analyses

For quantitative RT-PCR, total RNA was extracted from frozen leaves or cells with TRIzol (Invitrogen), according to the instructions from the manufacturer. The RNA was treated with 2 units of RNase-free DNase (Promega) and was further purified through RNeasy Mini Kit (QIAGEN) columns. First-strand cDNA was synthesized from 4 μg of total RNA using oligo-dT(18) and Transcriptase Reversa M-MLV (Invitrogen), according to the manufacturer's instructions.

Real-time RT-PCR reactions were performed as previously described [14]. To confirm the quality and primer specificity, we verified the size of the amplification products after electrophoresis through a 1.5% agarose gel and analyzed the Tm (melting temperature) of the amplification products by a dissociation curve, performed by the ABI7500 instrument. The primers used are listed in additional file 6. For the quantitation of the gene expression in the soybean protoplasts and seedlings, we used RNA helicase [14] as the endogenous control gene for data normalization in the real-time RT-PCR analysis. For the quantitation of the gene expression in tobacco leaves, we used actin as a control gene [[13]; ABI 158612]. The fold variation, which is based on the comparison of the target gene expression (normalized to the endogenous control) between experimental and control samples, was quantified using the comparative Ct method: 2^-(ΔCtTreatment - ΔCtControl)^. The absolute gene expression was quantified using the 2^-ΔCT ^method, and the values were normalized to the endogenous control.

### Transient overexpression in *Nicotiana tabacum *by Agrobacterium infiltration

Three- to four-week old tobacco leaves were infiltrated with Agrobacterium strain GV3101 pYFP-NAC6, as described [36]. Leaf segments (approximately 0.5 cm^2^) were excised from transfected leaves 3 days post-infiltration, and the protein expression was monitored by confocal microscopy. Leaf segments that displayed the visible appearance of cell death were collected, frozen in liquid nitrogen and stored at -80°C until use.

### Determination of chlorophyll content, lipid peroxidation and ion leakage

The total chlorophyll content was determined spectrophotometrically at 663 and 646 nm after quantitative extraction from individual leaves with 80% (v/v) acetone in the presence of approximately 1 mg of NaCO_3 _[37]. The extent of lipid peroxidation in the leaves was estimated by measuring the amount of MDA, a decomposition product of the oxidation of polyunsaturated fatty acids. The malondialdehyde (MDA) content was determined by the reaction of thiobarbituric acid (TBA), as described by Hodges et al. [38]. Electolyte leakage was measured from agroinoculated disc leaves as described by Wang et al. [39].

### Transient expression in protoplasts

Soybean protoplasts were prepared from 5-day-old sub-cultures of cotyledon cells of the soybean variety Conquista [40], as previously described [41], with some modifications. Briefly, the protoplasts were isolated five days after subculture by digestion for 3 h, under agitation at 40 rpm, with 0.5% cellulase, 0.5% macerozyme R-10, 0.1% pectolyase Y23, 0.6 M mannitol and 20 mM MES, pH 5.5. The extent of digestion was monitored by examining the cells microscopically every 30 min. After filtration through nylon mesh of 65 μm, the protoplasts were recovered by centrifugation, resuspended in 2 mL of 0.6 M mannitol plus 20 mM MES, pH 5.5, separated by centrifugation in a sucrose gradient (20% [w/v] sucrose, 0.6 M mannitol and 20 mM MES, pH 5.5) and diluted with 2 mL of electroporation buffer (25 mM HEPES-KOH (pH 7.2), 10 mM KCl, 15 mM MgCl_2 _and 0.6 M mannitol). Transient expression assays were performed by electroporation (250 V, 250 μF) of 10 μg of the expression cassette DNA and 30 μg of sheared salmon sperm DNA into 2 × 10^5 ^- 5 × 10^6 ^protoplasts in a final volume of 0.8 mL. Protoplasts were diluted into 8 ml of MS medium supplemented with 0.2 mg/ml 2, 4-dichlorophenoxyacetic acid and 0.6 M mannitol, pH 5.5. After 36 h of incubation in the dark, the protoplasts were washed with 0.6 M mannitol plus 20 mM MES, pH 5.5 and frozen in liquid N_2 _until use. Protoplasts were also prepared directly from soybean leaves as described [42].

### Caspase 3-like activity and *in situ *labeling of DNA fragmentation (TUNEL)

Total protein was extracted from soybean cells 36 h post-electroporation. The caspase 3-like activity was determined using ApoAlert^® ^Caspase 3 Colorimetric Assay Kit (Clontech), according to the manufacturer's instructions, at pH 7.4. The substrate was DEVD-pNA and the inhibitor of caspase 3-like activity was the synthetic tetrapeptide DEVD-fmk supplied by the kit. Free 3'OH in the DNA was labeled by the terminal deoxynucleotidyl transferase-mediated dUTP nick end labeling (TUNEL) assay using the ApoAlert DNA Fragmentation Assay Kit (Clontech) as instructed by the manufacturer. Formaldehyde-fixed semi-protoplasted cells that had been transformed with 35S:GmNAC6 were permeabilized with 0.2% Triton X-100/PBS and TUNEL labeled. Samples were observed with a Zeiss LSM 410 inverted confocal laser scanning microscope fitted with the configuration: excitation at 488 nm and emission at 515 nm. After being labeled by TUNEL, the slides were rinsed with PBS for 5 min at room temperature and counterstained with 10 μg ml^-1 ^propidium iodide (PI) containing 0.5 μg ml^-1 ^DNase-free RNAse. As positive control, samples were treated with DNase1.

### GmNAC6 Promoter Reporter Constructs

A 1000-bp fragment of 5'-flanking sequences of the *GmNAC6 *gene http://www.phytozome.net/soybean, relative to the translational initiation codon, was amplified from soybean DNA with the primers promNAC6Fw (5'- GAATTCGTCATTTGATTTAAGG-3', to create an EcoRI site, underlined) and pNAC6Rv (5'- AGATCTTCCATGGTTGCCATAT-3', creating the underlined BglII site) and then cloned into the TOPO-pCR4 vector (Invitrogen). The GmNAC6 promoter fragment was then released from TOPO-pCR4 with EcoRI and BglII double digestions and inserted into the same sites of pCAMBIA1381Z to yield pNAC6::GUS (pUFV1255).

### GUS activity assays

The protein extraction and fluorometric assays for GUS activity were performed essentially as described by Jefferson *et al*. [43] with methylumbelliferone (MU) as a standard. For the standard assay, leaf discs were ground in 0.5 mL of GUS assay buffer (100 mm NaH_2_PO_4 _·H2O [pH 7.0], 10 mM EDTA, 0.1% [w/v] sarcosyl, and 0.1% [v/v] Triton X-100), and 25 μL of this extract were mixed with 25 *μ*L of GUS assay buffer containing 2 mM of the fluorescent 4-methylumbelliferone *β*-D glucuronide (MUG) as a substrate [44]. The mixture was incubated at 37°C in the dark for 30 min, and GUS activity was measured using a DYNA Quant 200 Fluorometer.

## Authors' contributions

JAQA carried out the experiments, the statistical analysis of the data and drafted the manuscript. MTBR and PABR assisted directly the qRT-PCR assays and the caspase 3-like activity experiment.GLR assisted the Agro-infiltration experiments. GCM assisted directly the TUNEL assay and ion leakage assay. EPBF designed the experiments and edited the manuscript. All authors have read and approved the manuscript.

## Supplementary Material

Additional file 1 **Leaf yellowing and necrotic lesions caused by *NRP-B *expression in tobacco leaves**. Leaf sectors were infiltrated with the indicated Agro-inoculum and photographs were taken at 8 days (8d), 10 days (10d) and 11 days (11d) after Agroinoculation. Intense chlorosis was first detected at 8 days post-Agro-infiltration. Click here for file

Additional file 2 **Membrane ion leakage of NRP-B (A) and GmNAC6 (B) Agroinfiltrated leaf sectors**. Leaf sectors were infiltrated with the indicated Agroinoculum and ion leakage was measured from leaf discs harvested at 8 days (NRP-B) and 5 days (GmNAC6) post-infiltration. LBA is the result of leaf sectors infiltrated with untransformed *Agrobacterium tumefaciens *strain LBA4404. Click here for file

Additional file 3 ***Pseudomonas syringae patovar tomato *(*Pst*) induces a hypersensitive response in soybean**. A bacterial suspension of *Pst *was infiltrated in the abaxial epidermis of soybean leaves. The picture was taken 24 h after inoculation. Click here for file

Additional file 4 **Kinetics of GmNAC6 and NRP-B induction during protoplasting procedures**. Soybean protoplasts were electroporated with the empty vector pMON921 and the expression of endogenous GmNAC6 and NRP-B was monitored by quantitative RT-PCR using helicase as an endogenous control for the indicated times after electroporation. Click here for file

Additional file 5 **Putative cis-regulatory elements on the GmNAC6 promoter region**. GmNAC6 sequences extend until the ATG (bold) translational initiation codon of *GmNAC6*. Numbers indicate the position relative to the translation start codon. Several putative cis-regulatory elements are indicated in colors. These include a putative TATA box (pink), an inverted CAAT box (bold), an ABA-responsive element (in purple), a binding site of OsBIHD1 (in green), four putative elements (NGATT, in red) for the cytokinin-regulated transcription factor ARR1 and cis-elements (in blue) involved in response to dehydration stress and dark-induced senescence. Click here for file

Additional file 6 **Primers used for expression analysis by real time RT-PCR**. The table displays the sequence of the primers used for expression analysis of the indicated genes. The access numbers for the genes are also informed. Click here for file
