# Supplementary figures and images for: The NAC domain-containing protein, GmNAC6, is a downstream component of the ER stress- and osmotic stress-induced NRP-mediated cell-death signaling pathway
Source: BMC Plant Biol. 2011 Sep 26;11:129. doi: 10.1186/1471-2229-11-129 (PMC3193034; doi:10.1186/1471-2229-11-129)

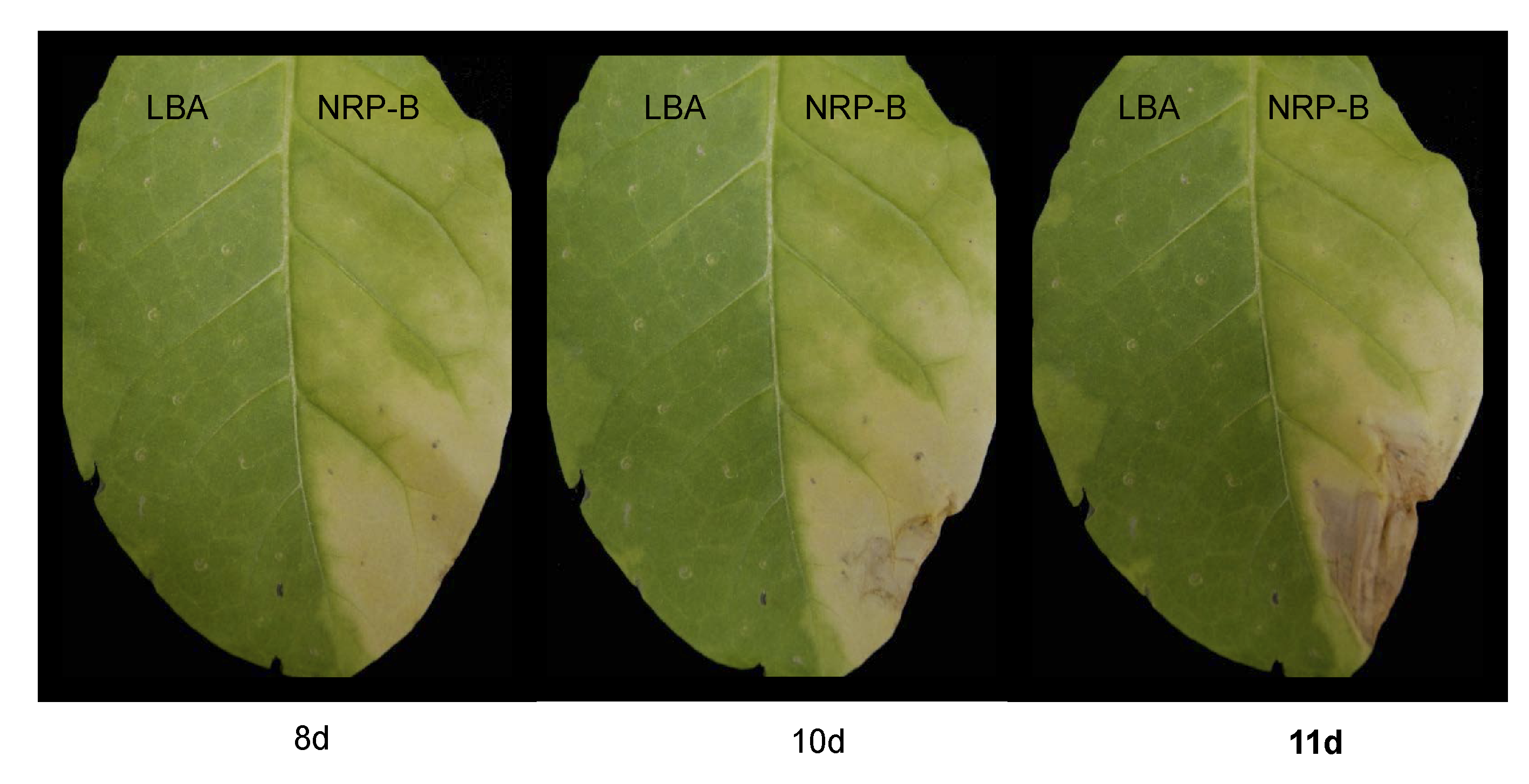

Supplement: Additional file 1 — Leaf yellowing and necrotic lesions caused by NRP-B expression in tobacco leaves. Leaf sectors were infiltrated with the indicated Agro-inoculum and photographs were taken at 8 days (8d), 10 days (10d) and 11 days (11d) after Agroinoculation. Intense chlorosis was first detected at 8 days post-Agro-infiltration. [file 1471-2229-11-129-S1.TIFF]

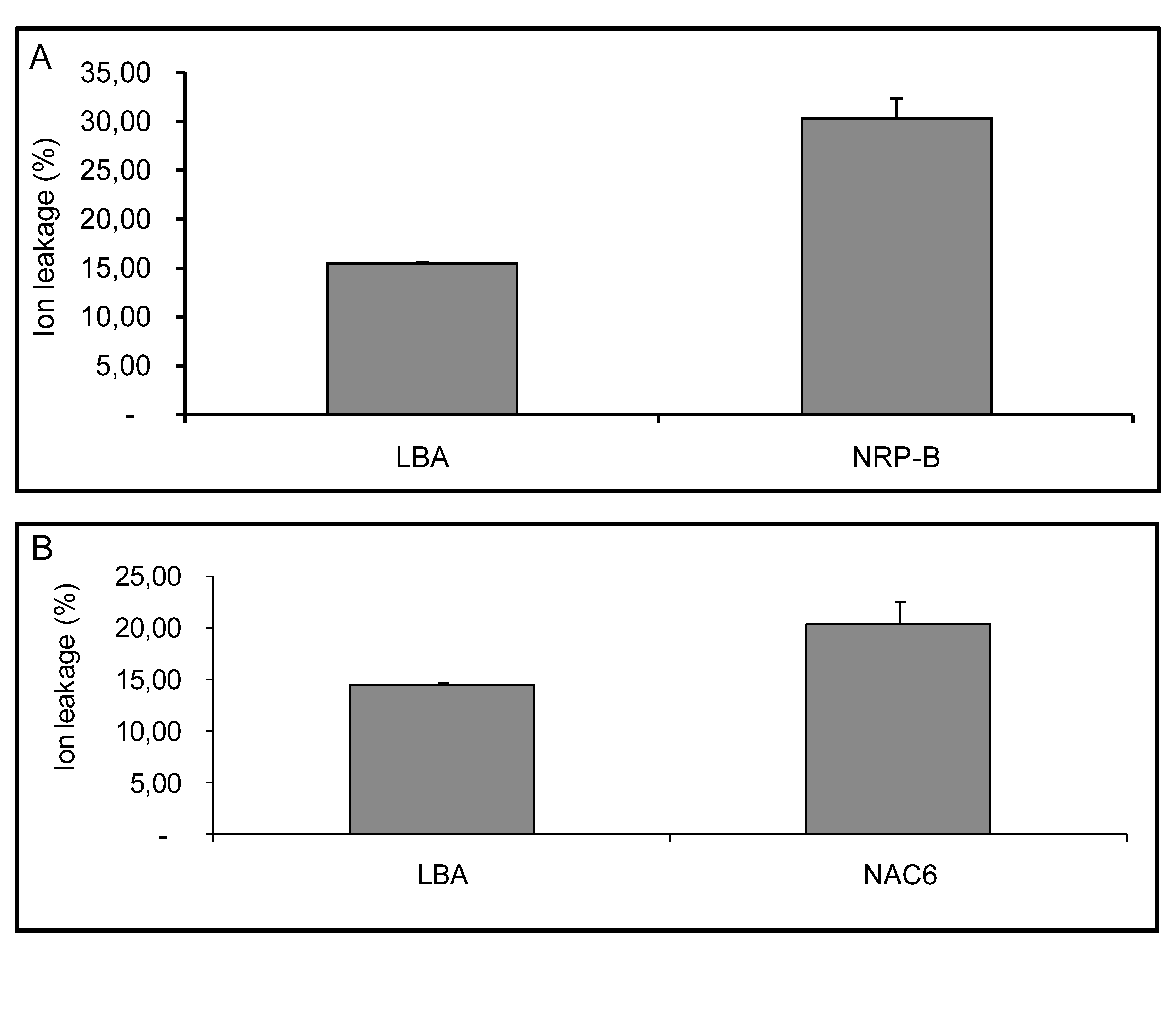

Supplement: Additional file 2 — Membrane ion leakage of NRP-B (A) and GmNAC6 (B) Agroinfiltrated leaf sectors. Leaf sectors were infiltrated with the indicated Agroinoculum and ion leakage was measured from leaf discs harvested at 8 days (NRP-B) and 5 days (GmNAC6) post-infiltration. LBA is the result of leaf sectors infiltrated with untransformed Agrobacterium tumefaciens strain LBA4404. [file 1471-2229-11-129-S2.TIFF]

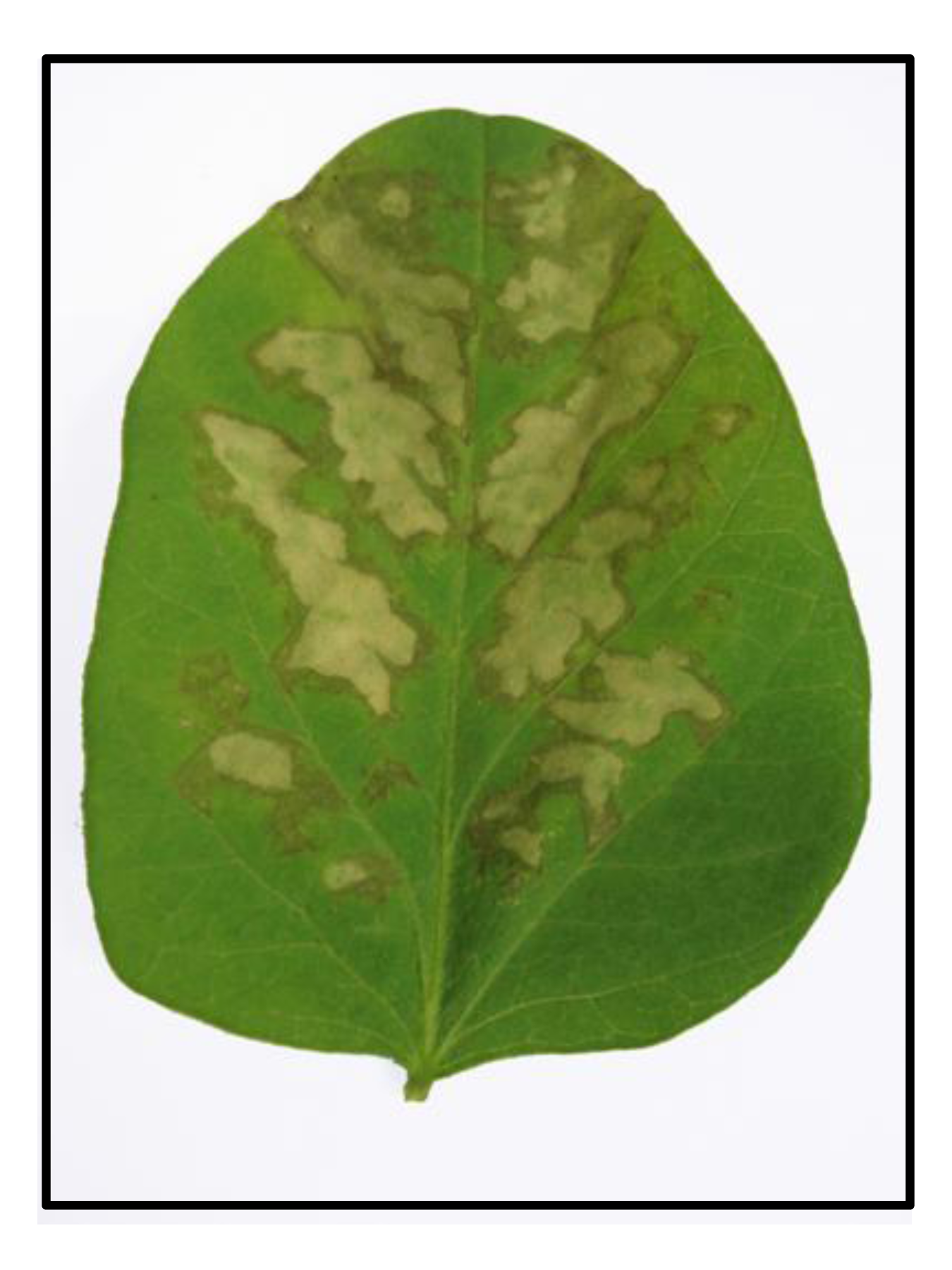

Supplement: Additional file 3 — Pseudomonas syringae patovar tomato (Pst) induces a hypersensitive response in soybean. A bacterial suspension of Pst was infiltrated in the abaxial epidermis of soybean leaves. The picture was taken 24 h after inoculation. [file 1471-2229-11-129-S3.TIFF]

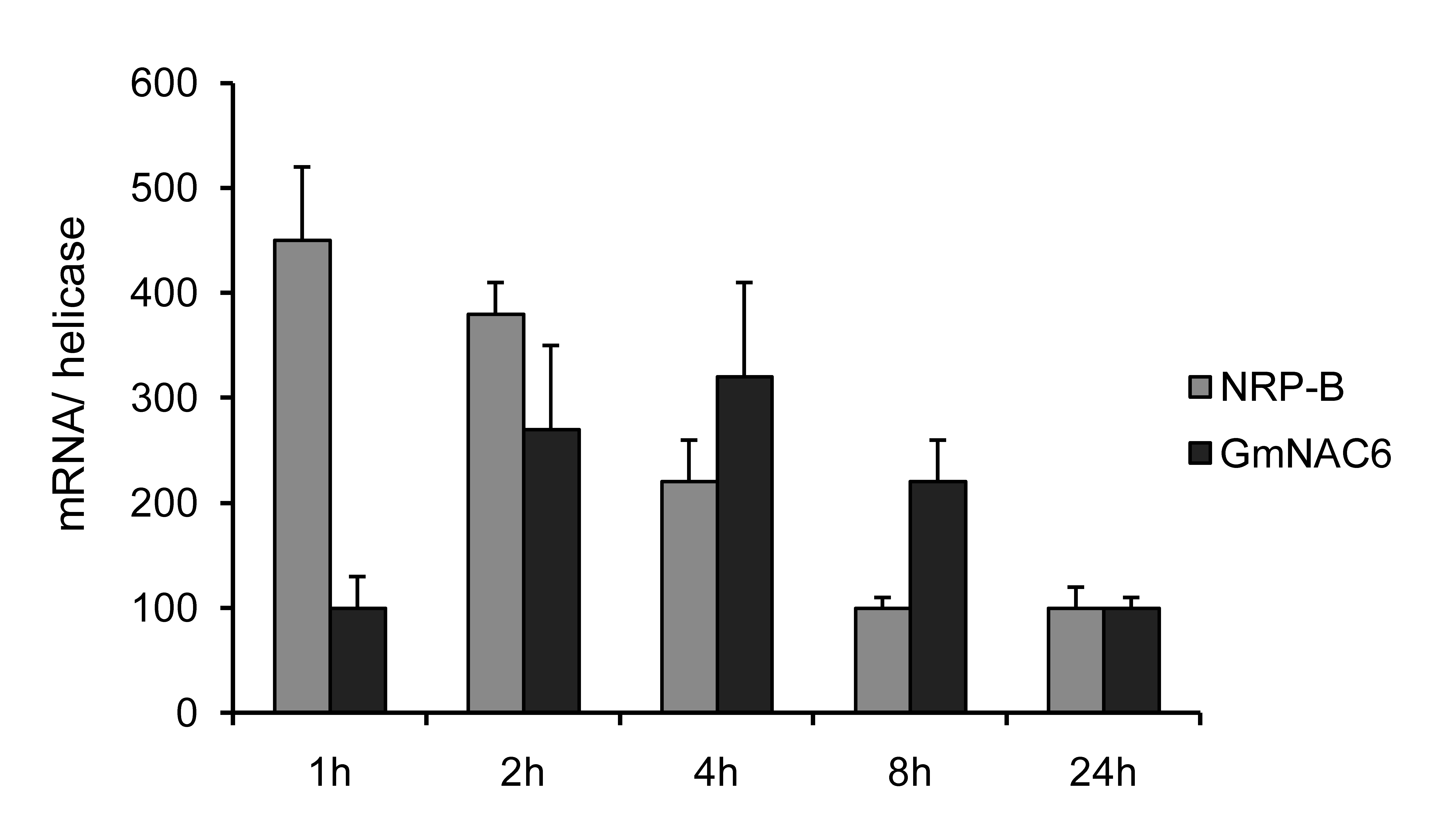

Supplement: Additional file 4 — Kinetics of GmNAC6 and NRP-B induction during protoplasting procedures. Soybean protoplasts were electroporated with the empty vector pMON921 and the expression of endogenous GmNAC6 and NRP-B was monitored by quantitative RT-PCR using helicase as an endogenous control for the indicated times after electroporation. [file 1471-2229-11-129-S4.TIFF]

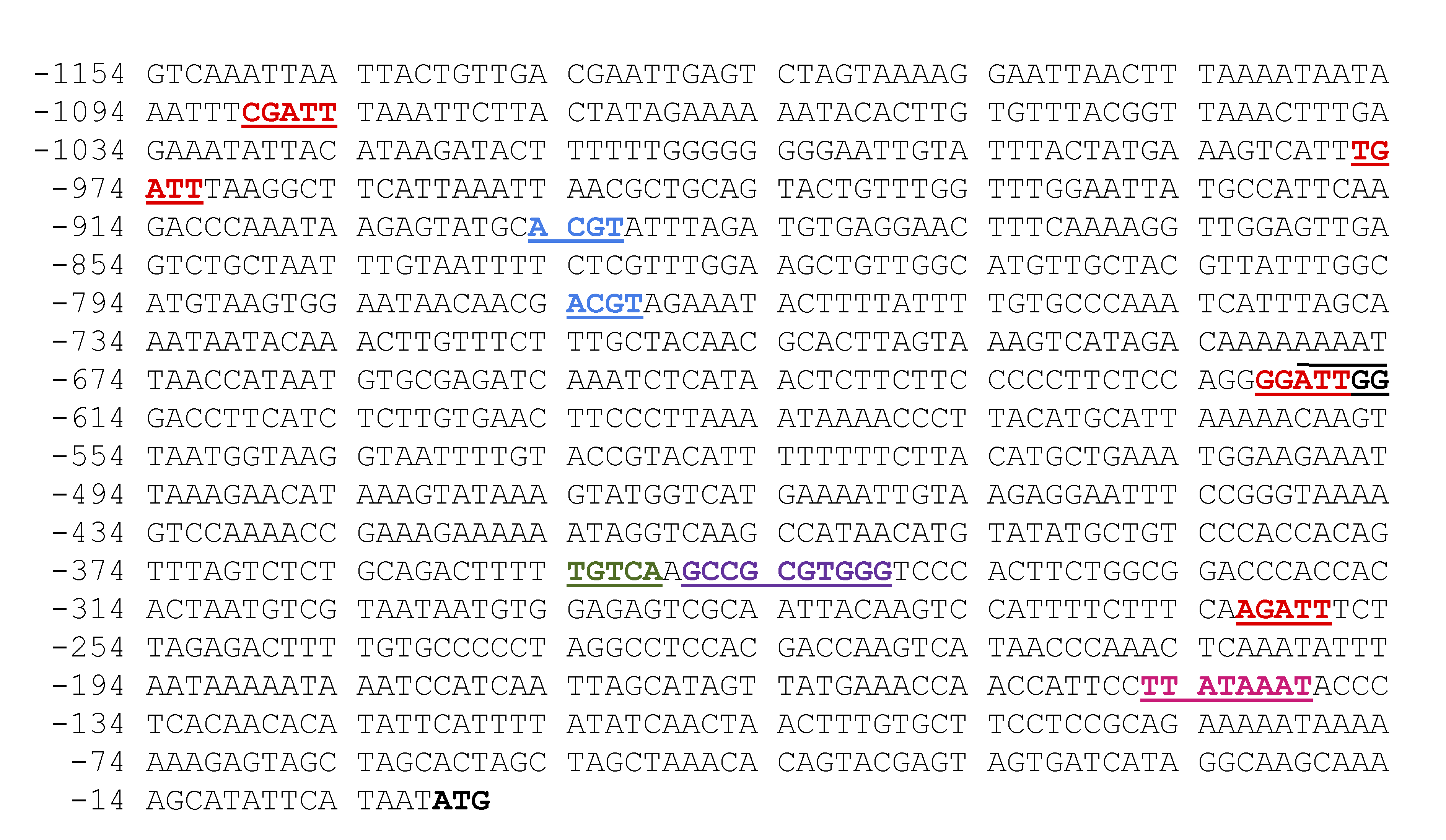

Supplement: Additional file 5 — Putative cis-regulatory elements on the GmNAC6 promoter region. GmNAC6 sequences extend until the ATG (bold) translational initiation codon of GmNAC6. Numbers indicate the position relative to the translation start codon. Several putative cis-regulatory elements are indicated in colors. These include a putative TATA box (pink), an inverted CAAT box (bold), an ABA-responsive element (in purple), a binding site of OsBIHD1 (in green), four putative elements (NGATT, in red) for the cytokinin-regulated transcription factor ARR1 and cis-elements (in blue) involved in response to dehydration stress and dark-induced senescence. [file 1471-2229-11-129-S5.TIFF]
