# Supplementary material for: The NAC domain-containing protein, GmNAC6, is a downstream component of the ER stress- and osmotic stress-induced NRP-mediated cell-death signaling pathway
Source: BMC Plant Biol. 2011 Sep 26;11:129. doi: 10.1186/1471-2229-11-129 (PMC3193034; doi:10.1186/1471-2229-11-129)
Supplement: Additional file 6 — Primers used for expression analysis by real time RT-PCR. The table displays the sequence of the primers used for expression analysis of the indicated genes. The access numbers for the genes are also informed. [file 1471-2229-11-129-S6.PDF]

Table S1- Primers used for expression analysis by quantitative real time RT-PCR (qRT-PCR)

Oligonucleotide	Seqüence (5' – 3')	Gene	Access number
HELIC Fw	TAACCCTAGCCCCTTCGCCT	Helicase	AI736067
HELIC Rv	GCCTTGTCGTCTTCCTCCTCG		
NAM Fw	ACGGAGACTTCAGATTCGGTGC	ATAF2	AW459852
NAM Rv	CATCGTTATTCCACTTGGGGTCGC		
Nac1 Fw	GGACTACCCAATAGCCCAAATCA	NAC1	AY974349 Gm0129x00049*
Nac1 Rv	GACCCAAGTAATCCATTTCCAAAAG		
Nac2 Fw	GGGTGCTTTGCCGTATTTACAA	NAC2	AY974350 Gm0026x00071*
Nac2 Rv	CTCCTCCGCTTTTCAGAATCTC		
Nac3 Fw	GAATGCAGCAATGGGTCATCA	NAC3	AY974351 Gm0027x00010*
Nac3 Rv	ATCCTGCTGGTGCATTGTTCTG		
Nac4 Fw	TGACCTCTATGTCCCTGCGTTA	NAC4	AY974352 Gm0178x00114*
Nac4 Rv	CCCCTGTGTGAAATCATTCTGA		
Nac5 Fw	CCCCAAAACTGCAAAGAATGAG	NAC5	AY974353 Gm0083x00164*
Nac5 Rv	GTGAGAGGTGGCAAAGCAGAAG		
Nac6 Fw	CCAACAAAAGCACTTGTGGCA	NAC6	AY974354 Gm0098x00246*
Nac6 Rv	GGACTATTCAACTGAGCCCAAAAG		
BiPD Fw	ATCTGGAGGAGCCCCAGGCGGTGG	BiPD	AF031241
BiPD Rv	CTTGAAGAAGCTTCGTCGTAAAACTAAG		
CALN Fw	TGATGGGGAGGAGAAGAAAAAGGC	CNX	AW508066
CALN Rv	CACTTGGGTTTGGGATCTTGGCTC		
NRP Fw	GGCACAAAGACTGGTGCTGAGA	NRP-A	AJ875407
NRP Rv	CTCTGTATCGTGGAGGCAGACC		
NRich Fw	TACAGGCATCCAATTTGGCGAACC	NRP-B	AI973541
NRich Rv	TGACTTGAAAGAGTTGATCTCACCCC		
GST Fw	CGGTTCTCATCCACAATGGCAAAC	GST	AAC18566
GST Rv	CAGCCCAGAATCTAGCCTGAGC		
GmPR1 Fw	AACTATGCTCCCCCTGGCAACTATATTG	PR1	BU577813
GmPR1 Rv	TCTGAAGTGGCTTCTACATCGAAACAA		
GmPR4 Fw	TGCGGGTGACAAATACAGGAA	PR4	AK246040
GmPR4 Rv	TGCTGCACTGATCTACGATTCTC		
NtActin Fw	AGCAAGGAAATTACCGCATTAGC	Actin	AB158612
NtActin Rv	ACCTGCTGGAATGTGCTGAGA		
NtPR1 Fw	CTGCTAAGGCCGTCGAGATGT	PR1	D90197
NtPR1 Rv	GAACCGAGTTACGCCAAACCA		
NtPR4 Fw	AGAGCGCCACAAACGTGAGAT	PR4	AW101647
NtPR4 Rv	GCCATGCGAGAGGCTTGTC		

* Corresponding access number to ID sequences Glyma0 (http://www.soybeangenome.org/documents/Glyma0.1.cds.fa.txt)
